# Impact of Environmental Chemicals on Lung Development

**DOI:** 10.1289/ehp.0901856

**Published:** 2010-05-05

**Authors:** Mark D. Miller, Melanie A. Marty

**Affiliations:** 1 Office of Environmental Health Hazard Assessment, California Environmental Protection Agency, Oakland, California, USA; 2 Pediatric Environmental Health Specialty Unit, University of California–San Francisco, San Francisco, California, USA

**Keywords:** cell signaling, children’s environmental health, developmental toxicology, lung development, lung disease, risk assessment, science policy

## Abstract

**Background:**

Disruption of fundamental biologic processes and associated signaling events may result in clinically significant alterations in lung development.

**Objectives:**

We reviewed evidence on the impact of environmental chemicals on lung development and key signaling events in lung morphogenesis, and the relevance of potential outcomes to public health and regulatory science.

**Data sources:**

We evaluated the peer-reviewed literature on developmental lung biology and toxicology, mechanistic studies, and supporting epidemiology.

**Data synthesis:**

Lung function in infancy predicts pulmonary function throughout life. *In utero* and early postnatal exposures influence both childhood and adult lung structure and function and may predispose individuals to chronic obstructive lung disease and other disorders. The nutritional and endogenous chemical environment affects development of the lung and can result in altered function in the adult. Studies now suggest that similar adverse impacts may occur in animals and humans after exposure to environmentally relevant doses of certain xenobiotics during critical windows in early life. Potential mechanisms include interference with highly conserved factors in developmental processes such as gene regulation, molecular signaling, and growth factors involved in branching morphogenesis and alveolarization.

**Conclusions:**

Assessment of environmental chemical impacts on the lung requires studies that evaluate specific alterations in structure or function—end points not regularly assessed in standard toxicity tests. Identifying effects on important signaling events may inform protocols of developmental toxicology studies. Such knowledge may enable policies promoting true primary prevention of lung diseases. Evidence of relevant signaling disruption in the absence of adequate developmental toxicology data should influence the size of the uncertainty factors used in risk assessments.

Over the past 20 years, acknowledgment of the unique vulnerabilities of children to the disruption of normal growth and development caused by environmental exposures has grown. The health impacts of many chemicals have been shown to differ based on the developmental window of susceptibility (e.g., periods of rapid cell proliferation or differentiation) when exposure occurs. Although the neurologic system has been most widely studied in this regard, there is a growing body of knowledge about the potential impacts of environmental exposures on lung growth and function. Respiratory disease has a large public health impact. An estimated 24 million U.S. adults have chronic obstructive pulmonary disease; 23 million have asthma; and chronic lower respiratory diseases rank as the fourth leading cause of death in the United States (Kung et al. 2008; National Heart, Lung, and Blood Institute 2007). In this article, we first present an overview of essential processes in lung development, followed by examples of xenobiotics, including important environmental contaminants, that can disrupt these processes.

Epidemiologic confirmation of the importance of the impact of early-life exposures, as originally described by Barker (the Barker hypothesis; Barker and Osmond 1986), has developed into a burgeoning field of study: the fetal origins of adult disease. Barker’s work demonstrating that poorer fetal nutrition and lower birth weight are associated with cardiovascular disease in adults has since been confirmed in multiple longitudinal studies around the world (Barker 2007). This finding was based on the observation that populations living in regions with poor fetal nutrition had higher risk of adult cardiovascular disease. Yet, paradoxically, geographical areas most associated with fetal or neonatal mortality and low birth weight (e.g., from poor nutrition) were not those at greatest risk for known postnatal risk factors for cardiovascular disease (e.g., high income, increased fat in diet). The premise for the early origins of adult disease is that during early life, “programming” in response to poor fetal nutrition results in permanent changes in organ structure, metabolism, and function. For example, lower birth weight has been associated with increased likelihood of having adult lipid profiles linked to cardiovascular risk as well as hypertension and impaired glucose regulation (Gluckman et al. 2008; Kajantie et al. 2008).

Although Barker’s original ecologic epidemiologic findings also showed a link between low birth weight and adult respiratory health, this was not accorded the same importance because geographic areas that had higher incidence of low birth weight and neonatal mortality were also regions with more postnatal risk factors (e.g., infection) for adult respiratory diseases. However, more recent studies provide evidence that *in utero* and early postnatal exposures set the stage for both childhood and later-life deficiencies in lung function that are predisposing factors for chronic obstructive lung disease and other disorders (Bush 2008; Canoy et al. 2007; Shi et al. 2007). For example, Stern et al. (2007) found that maximal expiratory flow at functional residual capacity measured in 123 infants at 2 months of age was associated with lung function measurements, including forced expiratory volume in 1 second (FEV_1_)/forced vital capacity (FVC), forced expiratory flow between 25% and 75% of FVC (FEF_25–75_), and FEV_1_, up to 22 years of age (Figure 1). In a longitudinal study of 243 infants, 4-week-olds with flow-limited tidal expansion (inability to increase expiratory flow with increased effort) were 7 times more likely to be diagnosed with asthma at 2 years of age (Young et al. 1994). These investigators observed a statistically significant increase in wheezing and a trend to reduced FEF_25–75_ at 11 years of age compared with children who had normal tidal expansion as infants (Turner et al. 2002). Similarly, airway responsiveness to histamine at 1 month of age was associated with abnormal airway function (decreased FEV_1_ and FVC), lower respiratory symptoms, and emergence of asthma by 6 years of age in a study of 95 children (Palmer et al. 2001). Several sizable studies from various countries have demonstrated that lung function in both asthmatics and nonasthmatics tracks from early childhood through adolescence and up to midlife, and is set by early-life events (Haland et al. 2006; Morgan et al. 2005; Oswald et al. 1997; Phelan et al. 2002; Stern et al. 2007). These studies support the importance of early-life programming of respiratory system structure and function and its life-long implications.

## The Endogenous Fetal Environment: Impacts on Fetal Respiratory Development

Evidence for the potential impact of the early-life environment on adult respiratory status stems from examination of the effects of fetal nutrition and sex. Intrauterine growth retardation (IUGR), often defined as low birth weight (< 10th percentile) for gestational age, has been identified as a risk factor for impaired lung function in children (Ergaz et al. 2005; Kotecha et al. 2010; Nikolajev et al. 2008). The reduction in nutrients and oxygenation related to IUGR may interfere with structural development of the lung (Lipsett et al. 2006). In an animal model, IUGR induced in late gestation produced alterations in alveolar function, corresponding to rapid development of alveoli during this time period (Maritz et al. 2001). Alterations include a thickened alveolar blood–gas barrier that persists into adulthood. In epidemiologic studies, birth weight has been demonstrated to be related to reduced lung function in adults (Canoy et al. 2007; Lawlor et al. 2005; Stein et al. 1997), and IUGR has been associated with poorer lung function at 8–9 years of age even when accounting for catch-up growth (Kotecha et al. 2010).

Endogenous chemicals, including estrogens and androgens, are associated with modulation of lung development and function (Carey et al. 2007). Both estrogen and androgen receptors are expressed in the human lung during fetal development and play a role in sexually dimorphic differentiation. For example, in humans, surfactant production and maturation appear earlier in females and may be a reason that males are more prone to respiratory distress syndrome (caused by surfactant deficiency) (Fleisher et al. 1985; Perelman et al. 1986). Androgens inhibit surfactant production via alterations in epidermal growth factor and transforming growth factor-β signaling (Dammann et al. 2000). In contrast, estrogen administration can stimulate surfactant production and lung maturation in the fetal rat and rabbit (Khosla et al. 1981). For premature infants, human male singletons or twins are at greater risk for respiratory morbidity and respiratory distress syndrome than are female singletons or twins. Shinwell et al. (2007) conducted a population-based study of 8,858 very-low-birth-weight premature infants to examine mixed-sex premature twins. Female infant respiratory morbidity was comparable with that of males, suggesting that a male disadvantage was transferred via an intrauterine paracrine mechanism to the female (Shinwell et al. 2007). Melamed et al. (2009) analyzed 2,704 twin births and found a similar disadvantage to the female in mixed-sex twins. Large-airway growth lags behind parenchymal growth in human males (relative to females), resulting in relatively narrower conducting airways (Becklake and Kauffmann 1999; Hoffstein 1986).

These examples show that the nutritional and endogenous chemical environments affect development of the lung and that these effects can be permanent, altering function in the adult. In this review we examine the basis for concern that similar adverse effects on lung development may occur after exposure to xenobiotics during critical windows in early life. We also discuss implications for regulation of environmental chemicals.

## Overview of Lung Development

The biology of lung development has been reviewed in many books and papers (e.g., Harding et al. 2004; Pinkerton and Joad 2000) and is briefly summarized here.

### Stages of lung development

**Figure 2:**
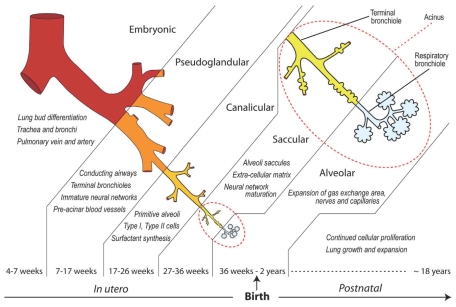
Principal stages of lung development in humans: diagrammatic representations of the timeline and developmental organization of trachea, primary bronchi, intrapulmonary bronchi, and acinus in the mammalian respiratory system. Reprinted from *Pharmacology and Therapeutics*, Vol 114 (Kajekar R. 2007. Environmental factors and developmental outcomes in the lung. Pharmacol Therap 114:129–145), copyright (2007), with permission from Elsevier.

In humans, primary lung buds develop during the fourth week of gestation from the endoderm of the foregut. After early embryonic development, stages in prenatal lung development are pseudoglandular, canalicular, saccular, and alveolar, which are represented in Figure 2, along with associated developmental features (Kajekar 2007). Only a portion of maturational events are required prenatally for successful survival, with most continuing postnatally during alveolarization (Pinkerton and Joad 2000).

The embryonic period is characterized by initial outpouching of primary bronchi from the primitive gut, which elongate into the mesenchyme and divide into two main bronchi. During the pseudoglandular phase (6–16 weeks of gestation in humans), branching continues and mesenchyme differentiates into cartilage, smooth muscle, and connective tissue around the epithelial tubules. By the end of this time, major conducting airways to the terminal bronchioles are developed. Respiratory bronchioles, which end in thin-walled dilatations (terminal sacs or primitive alveoli), develop in the canalicular period along with a rich vascular supply. During the saccular phase, first contact between the air space and proliferating pulmonary capillaries takes place. Epithelial cells differentiate into type I (thin) and type II (cuboidal). During alveolarization, the primitive alveoli develop secondary septa that form the walls of the true alveoli, resulting in a dramatic increase in surface area.

Cell types lining the conducting airway (trachea to midsize bronchioles) include ciliated cells, goblet cells (which produce mucus), and basal cells (stem cells for other cell types). The respiratory bronchioles include alveoli periodically along their surface. When the alveoli begin to dominate the surface area, they are termed alveolar ducts. Type I alveolar epithelial cells (AECs) are predominant in the alveolar wall and are associated with gas exchange. Type II AECs, which become mature during the alveolar stage, are the source of pulmonary surfactant. Premature infants lack sufficient numbers of differentiated type II AECs, often resulting in respiratory distress syndrome from inadequate production of surfactants. Alveolarization continues from late gestation (about 30 weeks) through at least the first 2 postnatal years. Growth of the lung continues through late adolescence (Gauderman et al. 2004).

There are > 40 morphologically differentiated cell types in the mature lung (Warburton et al. 1998). The development of each of these differentiated cell types is influenced by the spatial and temporal distribution of a variety of signaling molecules and their receptors, which regulate normal morphologic structure and function.

### Branching morphogenesis

Repeatedly branched structures develop when there is a need to maximize the contact between a structure and the surrounding environment. This organizational strategy packs a large area of contact into a small space (Davies 2006) and is a highly conserved process for organ growth in many structures, including the lung, kidney, salivary glands, prostate, and breast. Branching morphogenesis is a recurring, iterative patterning event of bud growth, bud elongation, and subdivision of terminal units (Cardoso 2004). In the lung, branching is used to generate the bronchial tree, including secretory glands, blood vessels, and interalveolar septa.

Mesenchyme develops from the mesoderm and gives rise to the lung’s connective tissue, endothelial cell precursors, smooth muscle that surrounds the airways and blood vessels, the lymphatic system, and the pleura. The lung develops in proximal-to-distal fashion but also in a specified right/left asymmetrical manner. These events develop in a cascade, each building on earlier activity and reiterated over several generations of branching to form the respiratory tree. Although many details of the regulation of these events are still unknown, numerous animal studies have illuminated many of the signaling pathways and transcription factors that direct lung development. Physical factors such as stretch and fluid volume also influence lung development.

Perhaps more important from a toxicologic perspective, reciprocal exchange of signaling factors between epithelial and mesenchymal cells is essential to normal development (reviewed by Cardoso and Lu 2006). For example, members of the fibroblast growth factor (FGF) family, soluble factors produced in the mesenchymal cells that signal tyrosine kinase receptors (Fgfr1–4) in the epithelial cells, are essential for bud initiation. Studies in organ culture show that the epithelium will migrate and proliferate toward Fgf10 (Park et al. 1998). Correct development of lung branching requires temporal and spatial control over Fgf10 levels (Bellusci et al. 1997; Mariani 2007). In rodents, deletion of *Fgf10* or lack of retinoic acid (RA), a crucial cofactor for FGFs, results in lung agenesis (Cardoso and Lu 2006; Desai et al. 2004; Sekine et al. 1999). Inhibitory control over Fgf10 involves sprouty (Spry2) and sonic hedgehog (Shh), which are highly diffusible factors secreted by the epithelium of the developing lung bud (Mason et al. 2006; Warburton et al. 2005). The Fgf10 in the distal mesenchyme diffuses into the epithelial bud and binds to the receptor, Fgfr2b, to induce budding and initiate branching (Bellusci et al. 1997). Fgfr2 activity induces Spry2, which inhibits FGF signaling and inhibits bud growth (Mailleux et al. 2001). Shh is highly expressed in the bud’s distal epithelium (Cardoso 2004). Shh from the bud is believed to progressively down-regulate FGF activity as the bud grows toward the Fgf10-expressing mesenchyme (Lebeche et al. 1999). In mice, overexpression of Spry2 or inhibition of Shh results in impairment of branching (Bellusci et al. 1997; Cardoso 2004; Lebeche et al. 1999; Mailleux et al. 2001).

Vascular endothelial growth factors (VEGF), necessary for development of the lung vasculature, are expressed in both branching tubular airways and vascular mesenchymal cells. The coordinated development of the epithelial and endothelial compartments depends on a VEGF gradient being present from its production at the tips of growing airway tubules (Voelkel et al. 2006). In a number of murine models, overexpression of *VEGF* results in dysmorphogenesis, and underexpression of *VEGF* or neutralization of VEGF results in poor septal formation and emphysematous changes (Gerber et al. 1999; Voelkel et al. 2006). These are only examples of the many signaling pathways that must be expressed in a precise temporal and spatial pattern for normal lung growth and development (Maeda et al. 2007).

The trachea develops from an outpouching of the foregut and requires formation of a separation between the two tissues. Mice deficient in Shh or RA or that lack *Nkx2.1*, the gene for a transcription factor active in early lung development [also called thyroid transcription factor 1 (*Ttf1*)], develop an incomplete separation of the foregut and trachea known as a tracheoesophageal fistula (Litingtung et al. 1998; Minoo et al. 1999). This is a relatively common congenital anomaly in humans and has been associated with down-regulation of the FGF pathways and aberrant Shh signaling (Crisera et al. 2000; Spilde et al. 2003).

### Alveolarization

As reviewed by McGowan and Snyder (2004), during the saccular phase the terminal portions of the airways give rise to alveolar ducts and sacs with thick walls of connective tissue and limited ability for gas exchange. The primary septa (the walls of the terminal sacs) have a central core of fibroblasts and connective tissue surrounded on each side by capillaries and epithelial cells. During alveolarization, ridges develop in the primary septa and become the secondary septa.

In humans, a period of rapid alveolar development lasts from about 36 weeks of gestation to about 2 years of age. From several months until 3 years of age, a second phase of alveolarization occurs, characterized by microvascular remodeling, thinning and lengthening of the secondary septae, loss of mesenchymal cells, and change from a dual to a single capillary bed. From birth until maturity, there is a 20‐fold increase in gas-exchange surface area (Burri 2006). Recent evidence in nonhuman primates suggests that alveoli increase in number through young adulthood (Hyde et al. 2007). If confirmed, it would expand the sensitive time period during which disruption of molecular mechanisms of alveolar development is of special concern, from the first few years of life until termination of longitudinal growth.

### Cellular differentiation and repair

With > 40 distinct cell phenotypes represented in the mature lung, undisturbed cellular differentiation is important to future lung function. In general, differentiation occurs in a proximal to distal sequence, with the tracheal epithelial cells differentiating first. Thus, identical critical processes involving cell signaling in development are occurring at different times, depending on the location proximal to distal in the respiratory tree.

Cross-talk between endothelial cells and epithelial cells is essential for differentiation of these cell types. VEGF-A expressed by the lung epithelium is essential to pulmonary capillary development. In mice, deficient capillary development by selective inactivation of the *Vegf-A* gene in the respiratory epithelium results in disruption of primary septae formation during alveolarization (Gerber et al. 1999; Yamamoto et al. 2007). Yamamoto et al. (2007) also demonstrated that hepatocyte growth factor (*Hgf*) expression in the developing endothelium is essential for normal epithelial development.

The transcription regulatory protein GATA binding protein 6 (GATA-6), expressed in lung epithelial cells, interacts with TTF1. In mouse models, inhibition of GATA-6 during fetal life inhibited terminal differentiation of respiratory epithelial cells (Liu et al. 2002; Yang et al. 2002). Elevated levels of GATA-6 postnatally (when levels normally are decreased) resulted in alterations in alveolar septation causing increased lung volumes and air space enlargement that persisted into adulthood (Liu et al. 2003). Airway resistance and airway and tissue elastance were significantly decreased. This provides an example of morphogenesis dependent on the precise timing of expression of GATA-6. Few studies have examined the impact of environmental chemicals on GATA-6 expression.

Clara cells are nonciliated bronchiolar cells and are the principal epithelial cells in the distal airway, whose products include Clara cell secretory protein and some components of surfactant. Clara cells are important in the metabolism of airborne toxicants due to their high cytochrome P450 (CYP450) content and are particularly sensitive to injury by xenobiotics, in part as a result of their ability to transform xenobiotics to reactive intermediates (Massaro et al. 1994). Clara cells are considered multipotent progenitor cells that regenerate airway epithelium after oxidant injury (Evans et al. 1976).

## Xenobiotic Disruption of Lung Development

Chemical exposures that affect expression of important growth regulators can result in more severe effects or even lethality when the exposure occurs during susceptible periods of lung development. Table 1 summarizes these cellular and subcellular impacts, along with associated alterations in lung structure or function (and possible clinical implications) for individual chemicals discussed here. Potential windows of susceptibility for exposure can be inferred by comparing the information in Table 1 with the timeline of key developmental periods in humans shown in Figure 2. Below we describe examples of xenobiotics that alter lung development by disrupting essential processes such as branching morphogenesis, alveolarization, and cellular differentiation and repair.

### The congenital diaphragmatic hernia model and nitrofen

Congenital diaphragmatic hernia (CDH) is a serious condition in newborns, with an incidence of about 1 in 3,000 live births in the United States. Many die *in utero*; morbidity and mortality in the first days after birth are high (Hartman 2004). Although CDH is associated with various genetic syndromes, recent understanding of its etiology has been changing. Originally it was believed to be due to malformation in a portion of the diaphragm, which allowed compression of the developing lung by abdominal contents entering the chest cavity, resulting in the subsequent characteristic hypoplastic lung. More recently, evidence has supported a “dual hit” theory which postulates that the original injury occurs early in lung development before and not connected to aberrant development of the diaphragm (Keijzer et al. 2000). According to this explanation, the already hypoplastic developing lung is then further inhibited as a result of the mechanical compression on the ipsilateral side resulting from herniation of the intestines into the thoracic cavity.

Exposure to nitrofen (2,4-dichlorophenyl-*p*-nitrophenyl ether), a banned pesticide, has been used as a model for CDH in rodents. Using this model, Leinwand et al. (2002) showed that the initial event in experimentally induced CDH is the development of hypoplastic lungs, which occurred early in development before the closure of the diaphragm and herniation. Therefore, compression was not the initial cause of hypoplasia. Hypoplasia has also been observed after nitrofen exposure in animals without CDH (Cilley et al. 1997). Lungs of nitrofen-exposed pups had 30% fewer terminal bronchioles than did controls, and they were developmentally immature (Leinwand et al. 2002). Similar observations in human infants with CDH include hypoplastic lungs, fewer alveoli, thickened alveolar walls, increased pulmonary interstitial tissue, and less airspace, as well as fewer bronchioles and vascular branches (Gallot et al. 2005).

The list of effects induced by nitrofen on various signaling pathways related to branching morphogenesis and lung development has been rapidly increasing. Wnt (wingless signaling proteins) growth factor signaling has been shown to have a role in regulation of proliferation, differentiation, and lineage specification during embryonic development. In the lung, *Wnt7* inactivation results in decreased branching and subsequent hypoplasia, as well as decreases in smooth muscle (Pongracz and Stockley 2006; Shu et al. 2002; Wang et al. 2005). Wnt signaling is an upstream regulator of bone morphogenetic protein 4 (BMP4) and FGF, both important in lung development. *Wnt7*-null mice die at birth from severe lung hypoplasia. In mice treated with nitrofen, *GATA‐6* (an upstream activator of *Wnt7b*) *Wnt7b*, *Wnt2*, and *BMP4*, were down-regulated (Takayasu et al. 2007a). GATA‐6, a zinc finger transcription protein, is an important regulator of distal epithelial cell differentiation, as well as proximal airway development (Yang et al. 2002). Prenatal RA partially mitigated the actions of prenatal nitrofen exposure in nitrofen-induced CDH rats (Montedonico et al. 2006, 2008).

Vitamin A–deficient diets have been linked to CDH in animal studies (Andersen 1941), and a small human epidemiologic study found lower levels of retinol (the active metabolite of vitamin A) in newborns with CDH than in controls (Major et al. 1998). Knockout mice deficient in RA nuclear receptors had an increased incidence of a spectrum of pulmonary agenesis, hypoplasia, and CDH (Mendelsohn et al. 1994). Nitrofen has been shown to disturb the RA signaling pathway at an early stage of lung development (Nakazawa et al. 2007a), and the incidence of CDH was dramatically reduced when RA was given along with nitrofen during pregnancy in rats (Babiuk et al. 2004). In rat lung explants, RA significantly increased the growth, number of lung buds, and lung area of nitrofen-induced hypoplastic lungs but had no effect on controls (Montedonico et al. 2006). One possible mechanism of nitrofen’s action on retinol may be to interfere with its cellular uptake during lung morphogenesis (Nakazawa et al. 2007b). Previous studies have suggested activity by inhibiting retinal dehydrogenase 2 (RALDH2), a key enzyme for generation of RA from retinal. Four chemicals shown to be able to precipitate development of CDH in animal models (nitrofen, bisdiamine, 4-diphenyl carboxylic acid, and SB210661) all have been found to inhibit RALDH2 (Mey et al. 2003).

One of the key processes in later gestation is the differentiation of a portion of alveolar type II cells to alveolar type I cells. That process was impaired in the nitrofen-induced CDH lung (Takayasu et al. 2007b). Although it appears that the immediate cause of decreased differentiation into type I cells was mechanical compression, this can be considered a secondary effect of the chemical exposure and was not observed in the absence of nitrofen exposure.

### Thyroid-disrupting chemicals and branching morphogenesis

Thyroid hormone is important for normal lung development (Van Tuyl et al. 2004). For example, alveolar septation, a largely postnatal structural manifestation, was impaired in hypothyroid mouse pups (Van Tuyl et al. 2004). The ratio of surfactant protein mRNA expression to that of corresponding proteins was influenced by both prenatal and postnatal thyroid hormone deficiency. A reduced ratio is indicative of an immature lung.

Nitrofen is a diphenyl ether and, like related chemicals, has antithyroid activity (Brandsma et al. 1994), inhibiting triiodothyronine (T_3_) receptor binding. Little research has been published on the effect of other environmental chemicals with antithyroid activity on lung development. Dioxins, polychlorinated biphenyls (PCBs), and polybrominated diphenyl ethers are structurally similar to nitrofen and have known antithyroid activity. A significant increase in the incidence of “bronchitis” was noted in a Taiwanese cohort of children exposed to PCBs prenatally (Rogan et al. 1988), which may involve PCB impairment of immune function and/or lung development. In an epidemiologic study in the Netherlands, ten Tusscher et al. (2001) found prenatal/lactational exposure to dioxins to be related to a significant reduction in lung function in 41 healthy children between 7 and 12 years of age. Gestational exposure in rats to 2,3,7,8-tetrachlorodibenzo-*p*-dioxin (TCDD) resulted in up-regulation of aryl hydrocarbon receptor (AhR) signaling in the developing lung and delayed lung development as evidenced by decreased total lung airspace and increased septal area (Kransler et al. 2009). These hypoplastic changes in lung morphology were associated with functional differences in respiratory mechanics. The study suggests that AhR activation adversely affects lung development. Up-regulation of AhR activity results in decreases in thyroid hormone due to increased metabolism. Thus, decreased thyroid hormone may play a role in these findings.

We identified no other studies that examined developmental exposure to thyroid-disrupting environmental chemicals and lung function. Lung function studies are a sensitive, minimally invasive method of measuring impact on lung development. Measurements of lung function in experimental animal pups would provide additional useful information on adverse lung impacts in developmental toxicity studies. In addition, more epidemiologic studies evaluating lung function in people exposed prenatally and postnatally to thyroid-hormone–disrupting chemicals are needed.

### Examples of disruption of branching morphogenesis by environmental chemicals in other organ systems

Branching morphogenesis is an essential developmental process for many organs that use common signaling pathways. One of the organs that exhibit branching morphogenesis is the salivary gland. TCDD exposure of cultured murine submandibular glands reduced epidermal growth factor signaling. This aryl hydrocarbon–associated effect inhibited branching morphogenesis and resulted in smaller glands with enlarged buds (Kiukkonen et al. 2006). This effect was seen in cells cultured at gestational day (GD) 13 but not later in development, indicating a specific window of susceptibility. Development of the prostate in the mouse is also inhibited by exposure to TCDD during pregnancy and lactation. Exposure on GD13 to a single dose resulted in reduced organ weight related to an inhibition of development of prostatic epithelial buds (Ko et al. 2002). Inhibition of prostatic bud development by TCDD is not androgen dependent (Lin et al. 2004).

TCDD exposure during a critical window of gestation produced disruption of branching morphogenesis in the rat mammary gland, resulting in smaller glands, limited branching, decreased numbers of terminal end buds, and a lack of substantial alveolar lobule development independent of hormonal effects (Fenton et al. 2002; Vorderstrasse et al. 2004). This effect was dependent on the time of exposure: It was observed after exposure on GD15 but not GD20, and persisted at least until postnatal day 45 (postpubertal) (Fenton et al. 2002).

Retinoids are involved in embryonic kidney patterning and development, including branching morphogenesis, and are important in kidney development. The Ret receptor modulates ureteric bud branching morphogenesis (Gilbert 2002). Nitrofen exposure resulted in hypoplastic kidneys in rats (Montedonico et al. 2007).

Exposure of rats *in utero* to the herbicide atrazine has been associated with altered branching morphogenesis in the mammary gland, although little detail is known of the exact mechanism of action (Rayner et al. 2005). Similarly, the mammary glands of offspring exposed during GD17–GD19 displayed delay in development of mature gland structures and less epithelial branching. Importantly, the offspring of atrazine-treated dams were unable to provide adequate nutritional support for the F_2_ offspring, resulting in decreased pup weight gain.

Clearly, the impact of exposure during critical windows of development to environmental chemicals that disrupt branching morphogenesis has been demonstrated in various organ systems dependent on this process. Chemicals that inhibit this process in one organ should be investigated for similar effects on lung development.

### Nicotine and alveolarization

The glycolytic pathway is very active during the alveolar phase of lung development and provides energy and precursors to the lung. Maternal exposure to nicotine in the rat resulted in sustained or permanent suppression of glycolysis and glycogenolysis in the lung tissue of the pup (Maritz 1986, 1988), due to reduced synthesis of phosphorylase and phosphofructokinase (the rate-limiting step in glycolysis) in nicotine-exposed animals (Kordom 2004; Kordom et al. 2002; Maritz 2004). As a result of inhibited glycolysis, Na^+^/K^+^-ATPase is inhibited, which may result in swelling and bleb formation of alveolar type I cells (reviewed by Maritz 2008).

In rats, maternal dosing with nicotine during pregnancy and lactation, at doses that did not alter fetal growth, resulted in a significant decrease in number of alveoli and increase in the alveolar volume at maturity in nicotine-exposed pups relative to controls (Maritz 2002; Maritz and Windvogel 2003). The increase in alveolar volume in nicotine-exposed pups was attributed to slower alveolar septal formation, flattening of the alveoli as they aged, and disappearance of alveolar walls leading to larger alveoli. The histopathology of nicotine-exposed animals resembled early emphysema (Maritz and Windvogel 2003). Routine pathologic examination of these lungs would likely fail to demonstrate these relatively subtle changes. Such irreversible changes could result in dysfunction (emphysema) in later life.

### Xenobiotics and cell differentiation and repair

In the rabbit, Clara cells actively differentiate postnatally, reaching maturity at 4 weeks. Despite lower levels of CYP450 activity in immature neonatal Clara cells, these cells were more susceptible to injury at lower doses of 4‐ipomeanol than adult cells (Plopper et al. 1994). Smiley-Jewell et al. (2000) treated rabbits with ipomeanol during three postnatal time periods that represent early, intermediate, and final stages of Clara cell maturation. In rabbits treated with a single dose of 4‐ipomeanol during early or middle periods of differentiation (up to 9 days of age), bronchiolar cell differentiation and repair were inhibited. The period of greatest susceptibility was during mid-differentiation at 7 days of age. On examination at 6 weeks, rabbits treated at day 7 had more squamous cells, fewer ciliated cells, reduced expression of Clara cell markers, and undifferentiated cuboidal cell ultrastructure compared with those treated at adulthood. In those treated at later stages of development (21 days), repair was completed during this time period, and the bronchiolar epithelium was indistinguishable from controls at 6 weeks (Smiley-Jewell et al. 2000). Thus, the extent of repair was related to the stage of cellular differentiation at the time of injury but independent of the degree of injury (Smiley-Jewell et al. 1998, 2000). Exposure during the critical window of early development disproportionately inhibited differentiation and repair of bronchiolar Clara cells, resulting in persistent, possibly permanent effects.

Similarly, naphthalene, another bioactivated Clara cell toxicant, has been found to induce injury at much lower doses in immature mice (Fanucchi et al. 1997). 1‐Nitronaphthalene, an atmospherically formed nitroaromatic, more severely affects immature than mature Clara cells in both mice and rats (Fanucchi et al. 2004).

These findings suggest that Clara cell injury by bioactivated chemicals in the developing animal is not necessarily predicted by the level of CYP450 enzyme activity. There is no reason to believe this is restricted to mice, rats, and rabbits. Susceptibility of the neonate to injury cannot be predicted by the findings in adults. Early-life injury may be a precursor to dysfunction later in life.

## Widespread Environmental Chemicals That Disrupt Lung Development

### Ozone

Lung development occurs not only prenatally but through childhood, and lung function grows at least through adolescence. Therefore, exposure to environmental toxicants may affect underlying structural and functional aspects of the lung during a wide window. This may increase susceptibility to asthma as well as other diseases.

**Figure 3:**
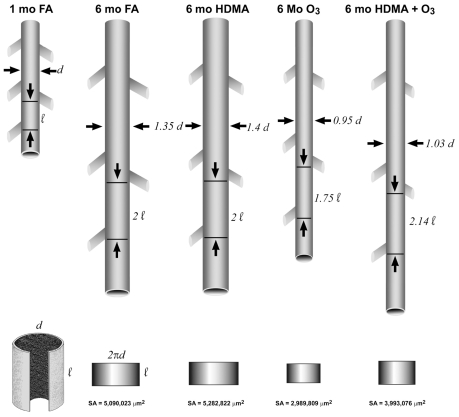
Diagrammatic comparison of differences in the size of one generation of distal bronchiole in the left cranial lobe of infant rhesus monkeys (180 days of age) following 11 cycles of exposure to filtered air (FA), HDMA, O_3_, or both HDMA and O_3_. The airway measured is the bronchiole proximal to the terminal bronchiole in the axial airway path of the caudal segment of the left cranial lobe of each animal. Relative scaling for length (*l*) and diameter (*d*) is based on setting the value for 30-day-old animals (when exposure began) equal to “*l*.” Reprinted from Plopper et al. (Plopper CG, Smiley-Jewell SM, Miller LA, Fanucchi MV, Evans MJ, Buckpitt AR, et al. 2007. Asthma/allergic airways disease: does postnatal exposure to environmental toxicants promote airway pathobiology? Toxicol Pathol 35:97–110), *Toxicologic Pathology* Vol. 35(1); copyright 2007; reprinted by permission of SAGE Publications.

An elegant series of studies conducted at University of California–Davis evaluated the impact of ozone (O_3_) and allergens on lung development in rhesus monkeys. These studies integrated early-life exposures through multiple windows of susceptibility to observe functional and structural changes relevant to human lung development and lung disease. Tran et al. (2004a) described conducting airway growth studies in monkeys, with airways doubling in length and increasing by 33% in diameter between 1 and 6 months of age. Postnatal exposure to O_3_, alone or combined with house dust mite antigen (HDMA), resulted in changes in bronchiolar growth patterns, inhibiting growth in diameter and promoting growth in length (Figure 3), as well as reducing the number of conducting airway branches (by as many as six generations) (Fanucchi et al. 2006; Plopper et al. 2007). These changes appeared to be permanent because they persisted after 6 months of recovery.

In monkeys, the smooth muscle bundles grow along with the conducting airways and have characteristic alterations depending on location and age (Tran et al. 2004a). Muscle bundles progress with age from a primarily perpendicular orientation relative to the long axis of the airway in early development to an orientation with a large percentage of bundles > 30° from perpendicular. This process is disrupted by exposure to HDMA or O_3_ in rhesus monkeys (Fanucchi et al. 2006; Tran et al. 2004b). The smooth muscle bundles were thickened, and their orientation relative to the airway in the respiratory bronchioles was altered by exposure to O_3_ such that a much higher percentage of the bundles were < 15° perpendicular to the long axis of the bronchiole, an alteration that may increase airway hyperreactivity (Plopper et al. 2007). The changes noted above from O_3_ exposure were not associated with changes in lung volume or function as measured by routine testing. The effect of the structural changes found after exposure to HDMA and O_3_ is consistent with alterations in airflow and resistance found in asthmatics (James and Carroll 2000; Plopper et al. 2007; Tran et al. 2004b). Epidemiologic evidence suggests that exposure to O_3_ is associated with asthma induction in children (McConnell et al. 2002) and reduced lung function growth (Galizia and Kinney 1999; Tager et al. 2005).

In infant rhesus monkeys, chronic cyclic O_3_ exposure (0.5 ppm, 8 hr/day, for 5 days, followed by 9 days of filtered air, for 11 cycles) induced biochemical and functional alterations (depleted proteoglycan and Fgf2, altered Fgfr1) and thinning of the basement membrane zone (Fanucchi et al. 2006). This tissue binds and releases growth factors, is involved in cell–cell communication, and functions as a barrier. The alteration of Fgf2 signaling, important for regulating processes in the developing lung, may be associated with the reported O_3_-induced abnormal development of alveolar and bronchiolar regions in animal models.

### Arsenic

Arsenic is a known human carcinogen, causing lung cancer after inhalation and oral exposure. Long-term arsenic exposure through drinking water has also been associated with chronic nonmalignant respiratory disease and decreased lung function (Guha Mazumder 2007). In men exposed in West Bengal, India, chronic ingestion of arsenic was a stronger determinant of lung function than was smoking, and decreases in FEV_1_, FVC, and FEF_25–75_ indicated restrictive pathophysiology involving the small airways (von Ehrenstein et al. 2005).

Several studies have demonstrated transplacental carcinogenesis in animal models. Waalkes et al. (2003) reported that *in utero* arsenic exposure in C3H mice resulted in tumorigenesis in a number of organs, including the lung. These investigators reported increased lung tumors in adult females, but not in males. In humans, exposure to arsenic via drinking water *in utero* and postnatally was associated with much larger increases in lung cancer and bronchiectasis in a population in Chile than were later-life exposures (Smith et al. 2006). Thus, arsenic exposure early in life is associated with both chronic obstructive lung disease and lung cancer, and data from animal models indicate that this may result from aberrant signaling during lung development.

Gestational arsenic exposure increased expression of the estrogen receptor-α gene (*ER-*α) and genes related to estrogen signaling in the female fetal mouse lung after environmentally relevant exposures via drinking water (Shen et al. 2007). This correlated to intense overexpression of ER-α protein in lung tumors in adult females exposed gestationally. These authors also observed activation of a number of genes associated with lung cancer development, including *EGFR* (epidermal growth factor receptor), *AFP* (α-fetoprotein), and *L‐myc* (lung associated myc oncogene) after gestational exposure.

The extracellular matrix in the lung performs various functions, including providing structural support for cells, regulating intercellular communication, and storing growth factors locally. Extracellular matrix fibers include collagen (structural support) and elastin (elasticity). Matrix genes were dysregulated by chronic arsenic exposure in adult mice (10 or 50 ppb), including down-regulation of the gene for collagen, elastin, and fibronectin and up‐regulation of the matrix-degrading enzyme MMP‐9 (Hays et al. 2008; Lantz and Hays 2006). *In vitro* exposure of human epithelial cells to arsenic also caused up‐regulation of MMP‐9 and restricted wound repair (Olsen et al. 2008). Collagen-knockout mice have increased smooth muscle around the airways (Dekkers et al. 2007; Parameswaran et al. 2006). Similarly, *in utero* and early postnatal exposure of mice to environmentally relevant levels of arsenic resulted in decreased total collagen in the airway adventitia and increased smooth muscle surrounding the airway at 28 days of age (Lantz et al. 2009). These mice exhibited airway hyperresponsiveness as increased bronchoconstriction in response to methacholine challenge. The structural alterations were not present in mice exposed to arsenic as adults. Because these irreversible findings were identified by immunohistochemistry and methacholine challenge, they would not likely be identified by standard toxicology studies.

Arsenic can disrupt the highly complex signaling between embryonic lung tissues of mesenchymal and endodermal origin and can permanently alter lung structure and function when exposure occurs at key developmental windows. Petrick et al. (2009) exposed pregnant rats to 500 ppb arsenic via drinking water starting at GD1 and evaluated gene expression in fetal lung on GD18. Lung weights were lower in arsenic-exposed pups than in controls. Arsenic exposure altered expression of key genes in pathways involved in lung development, including the β‐catenin pathway, which is required for proper cell migration during branching morphogenesis. Deletion of the β‐catenin gene in epithelial cells resulted in a lack of peripheral lung structures (Mucenski et al. 2003). Thus, altered signaling caused by arsenic exposure results in poor airway structure. Malformed airways are characteristic of bronchiectasis, which is observed in humans exposed to arsenic via drinking water.

### Di(2-ethylhexyl) phthalate

The phthalate ester plasticizers interact with the nuclear hormone receptor superfamily peroxisome proliferator–activated receptor (PPAR) and are thereby capable of modulating gene transcription in a number of tissues.

There are significant levels of PPARγ in human lung (Chang and Szabo 2000), and di(2-ethylhexyl) phthalate (DEHP) metabolites can bind to these receptors. Through the use of transgenic mice, epithelial cell PPARγ has been shown to be directly involved in lung maturation, probably through altering airway cell differentiation and resultant phenotype (Simon et al. 2006). Lungs of mice whose conducting airways are deficient in PPARγ develop abnormally and have enlarged airspaces and altered lung mechanics, possibly due to altered epithelial–mesenchymal interactions during development.

Rats exposed during the last week of pregnancy and first 2 days postnatally via oral administration of 1,000 mg/kg-day DEHP to the dams had abnormal lung histology (Magliozzi et al. 2003). Focal thickening of primary septa was evident, and there were more dilated air spaces in the parenchyma in DEHP-treated pups, resulting in a decrease in gas exchange surface. Type II pneumocytes, which are a major source of surfactant, were enlarged and more numerous in treated pups than in control pups. These authors noted that lung parenchymal changes were similar to that seen in children with chronic lung disease and in animal models of bronchopulmonary dysplasia. The histopathology suggests impaired alveolar maturation and not tissue degeneration. Magliozzi et al. (2003) also noted that type II pneumocytes were more numerous and larger in the DEHP-treated pups and that the peroxisomes in the alveolar type II pneumocytes of treated pups appeared unaffected; thus, they suggested that DEHP action in the fetal lung is not dependent on interaction with the PPARα. Rather, the effect might be due to interaction with PPARγ to alter surfactant production, secretion, or reabsorption by type II cells. PPARγ ligands have been shown to down-regulate surfactant protein B expression in alveolar type II cells (Yang et al. 2003). Rosicarelli and Stefanini (2009) reported that DEHP may affect alveolar development by disrupting the timing of epithelial and mesenchymal cell proliferation; they found that DEHP treatment of the dams was associated with both an impaired secretion pattern and altered proliferation rate of septal myofibroblasts. In a nested case–control study of 198 children with allergic symptoms and 202 controls, Bornehag et al. (2004) identified a statistically significant association between DEHP levels in household dust and doctor-diagnosed asthma. The interactions of the ubiquitous phthalate ester plasticizers such as DEHP with PPARγ during lung development need further study.

## Conclusion

The lung is susceptible to many influences during early development, including endogenous hormones, pharmaceuticals, and environmental chemicals. Chemical exposure during developmental windows may produce lifelong structural and functional alterations, and some may become apparent only later in life (e.g., as lung function naturally declines with age). Susceptible maturational events occur throughout prenatal development, postnatally, and through adolescence. Evidence is accumulating that clinically significant disruption of lung development may be caused by some xenobiotics at environmentally relevant doses (e.g., arsenic, O_3_). Nonetheless, there is a paucity of literature evaluating the impact of early-life exposure to environmental chemicals on lung structure and function.

Many fundamental biologic processes (e.g., branching morphogenesis) and associated signaling events involved in development of multiple organs are highly conserved. A variety of transcription factors and morphoregulatory molecules essential to these processes are susceptible to interference during critical developmental stages. Examples presented in this review highlight the potential of a chemical to affect development in multiple organs that use the same fundamental patterning and developmental building blocks. Although local tissue differences may alter the impact of signaling disruption, a chemical that is identified as having the potential to disrupt fundamental processes in one organ (e.g., dioxin disrupting branching morphogenesis in the prostate) should be evaluated appropriately for similar impacts in the lung. In addition, chemicals that are structurally similar (e.g., PCBs) to those known to affect the developing lung (e.g., nitrofen) should be evaluated with appropriate studies for their impact on lung development.

Studies to determine the potential toxicity of xenobiotics resulting from early-life exposures should incorporate knowledge of early signaling events into experimental protocols. Evidence from animal studies indicates that many lung alterations induced by environmental chemicals require functional and/or highly specific studies targeted at identifying alterations of structure or function. Many of the functionally significant impacts of early-life exposures on lung development we discussed here would not have been identified with standard toxicologic study protocols.

Risk assessment practice should use data on disruption of basic developmental processes to inform the size of applied uncertainty factors. When there is evidence that a chemical can disrupt relevant signaling pathways but developmental toxicity data are inadequate, uncertainty related to this data gap should be reflected in the assessment. For example, the risk assessor could increase the size of the intraspecies uncertainty factor used in noncancer risk assessment to account for increased sensitivity of early life stages.

The U.S. Environmental Protection Agency (EPA) Strategic Plan for Evaluating the Toxicity of Chemicals (U.S. EPA 2009) and the National Academy of Sciences report *Toxicity Testing in the 21st Century* (National Research Council 2007) envision a transformation in the approach to toxicity testing that focuses on “toxicity pathways.” Toxicity pathways are “cellular response pathways that, when sufficiently perturbed in an intact animal, are expected to result in adverse health effects” (National Research Council 2007). These cellular pathways include key signaling pathways in development. The U.S. EPA (2009) noted that “an inventory of toxicity pathways and their involvement in a variety of toxicological responses needs to be created.” We suggest that the key signaling pathways in branching morphogenesis (e.g., a highly conserved fundamental developmental process that uses similar signaling pathways across multiple organs) represent important toxicity pathways. Evidence that a chemical interferes with an important signaling event should inform decisions on relevant end points for studies of developmental toxicity and help identify chemical groupings for which a cumulative evaluation may be appropriate, because they affect the same toxicity pathway. Ultimately, knowledge of the impacts of xenobiotics on lung development can be used to develop policies promoting true primary prevention of chronic obstructive pulmonary disease, asthma, and other lung diseases.

## Figures and Tables

**Figure 1:**
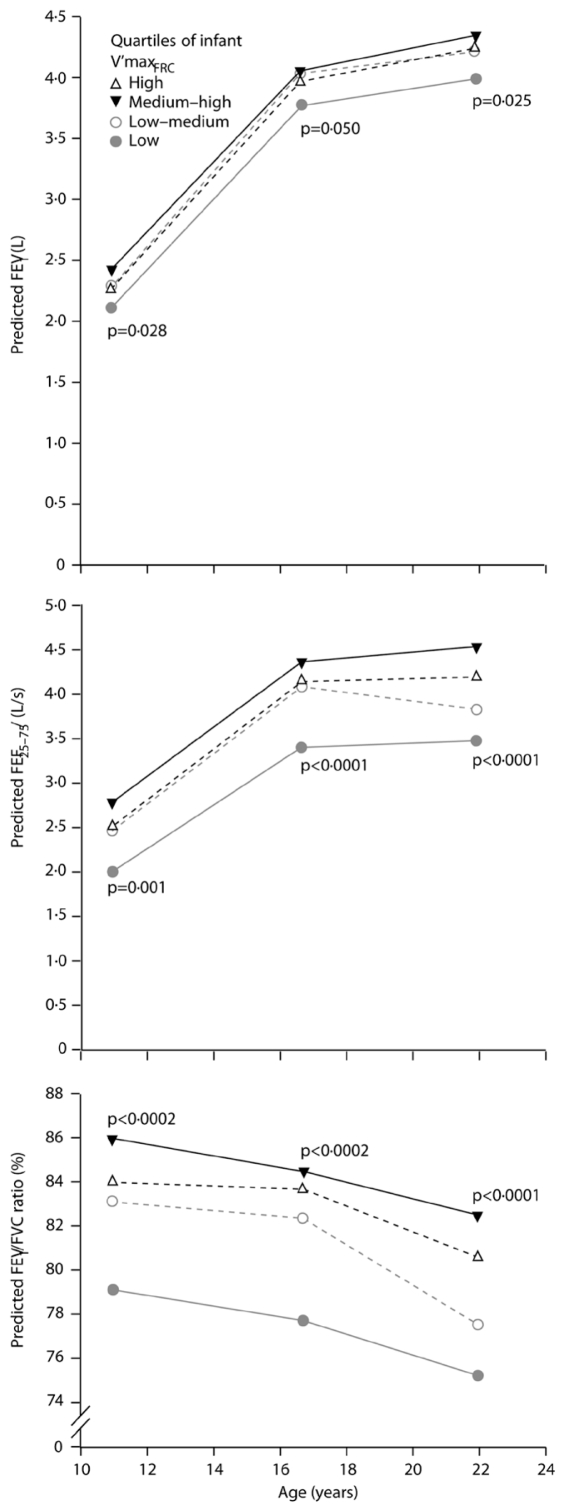
Predicted mean values for lung function in males at 11, 16, and 22 years of age by length-adjusted infant lung function, standardized to mean height and weight and measured as maximal expiratory flow at functional residual capacity (*V*_maxfrc_); 14% of variance in lung function of young adults was related to airway function at 2 months. Reprinted from *The Lancet*, Vol. 370 (Stern DA, Morgan WJ, Wright AL, Guerra S, Martinez FD. 2007. Poor airway function in early infancy and lung function by 22 years: a non-selective longitudinal cohort study. Lancet 370:758–764), copyright (2007), with permission from Elsevier.

**Table 1:** Cellular, structural, and functional impacts on lung development of xenobiotics.

Chemical	Cellular and subcellular level impacts	Structural or functional impact	Possible clinical implications	References
Nitrofen (2,4-dichlorophenyl-*p*-nitrophenyl ether)	Down-regulates GATA-6, Wnt7, BMP4, FGF, and RALDH2; decreases RA synthesis; inhibits T_3_ receptor binding	Decreased branching; altered smooth muscle, surfactant, and alveolar septation	Pulmonary hypoplasia, immature lung	Brandsma et al. 1994; Leinwand et al. 2002; Major et al. 1998; Mendelsohn et al. 1994; Montedonico et al. 2008; Pongracz and Stockley 2006; Shu et al. 2002; Takayasu et al. 2007a; Wang et al. 2005
TCDD	AhR, thyroid hormone	Delayed lung development, decreased total lung space, increased septal area	Chronic bronchitis,^a^ decreased functional capacity, chronic obstructive pulmonary disease (?)	Kransler et al. 2009; Rogan et al. 1988^a^; ten Tusscher et al. 2001
Nicotine	Suppresses glycolysis and glycogenolysis, reduces synthesis of phosphorylase and phosphofructokinase, inhibits Na^+^/K^+^-ATPase	Slower septal formation, bleb formation, decreased number of alveoli, increased alveolar volume	Decreased functional capacity, emphysematous changes	Kordom 2004; Kordom et al. 2002; Maritz 1988, 1986, 2002; Maritz and Windvogel 2003
4-Ipomeanol, naphthalene, 1-nitronaphthalene	Inhibits bronchiolar cell differentiation and repair	Injury/loss of Clara cells	Increased susceptibility to inhaled toxicants, alteration in surfactant	Fanucchi et al. 1997, 2004; Plopper et al. 1994; Smiley-Jewell et al. 1998, 2000
Ozone	Depletes proteoglycan and Fgf2, alters Fgfr1, thinning basement membrane zone	Altered bronchiolar growth (longer/decreased diameter), fewer branches, alters orientation of bronchiolar smooth muscle	Increased airway hyperreactivity,^a^ emphysema (?), decrease in lung function growth,^a^ asthma induction^a^	Fanucchi et al. 2006; Galizia and Kinney 1999^a^; McConnell et al. 2002^a^; Plopper et al. 2007; Tager et al. 2005^a^
Arsenic	Increases ER-α expression, dysregulates matrix genes; β-catenin up-regulates EGFR, L-myc, and AFP	Altered branching and cell migration, decreased elasticity and structural support	Bronchiectasis,^a^ airway hyperreactivity, lung cancer^a^	Guha Mazumder 2007^a^; Hays et al. 2008; Lantz et al. 2009; Petrick et al. 2009; Shen et al. 2007; Smith et al. 2006^a^; Waalkes et al. 2003; Von Ehrenstein et al. 2005^a^
DEHP	Binds to PPARγ, altering airway cell differentiation and surfactant protein production	Thickened primary septa, fewer/more dilated airspaces, increased type II pneumocytes	Bronchopulmonary dysplasia, altered lung mechanics, altered surfactant regulation, asthma^a^	Bornehag et al. 2004^a^; Chang and Szabo 2000; Magliozzi et al. 2003; Rosicarelli and Stefanini 2009; Yang et al. 2003;

Abbreviations: AFP, α-fetoprotein; AhR, aryl hydrocarbon receptor; BMP4, bone morphogenetic protein 4; EGFR, epidermal growth factor receptor; ERα, estrogen receptor-α; L‐*myc,* lung associated myc oncogene; PPARγ, peroxisome proliferator–activated receptor γ; RALDH2, retinal dehydrogenase 2; T_3_, triiodothyronine.

a Includes evidence in humans.
